# A cluster randomized trial of utilizing a local change team approach to improve the delivery of HIV services in correctional settings: study protocol

**DOI:** 10.1186/2194-7899-1-8

**Published:** 2013-12-23

**Authors:** Steven Belenko, Christy Visher, Michael Copenhaver, Matthew Hiller, Gerald Melnick, Daniel O’Connell, Frank Pearson, Bennett Fletcher, the HIV-STIC Workgroup of CJDATS

**Affiliations:** 1 Temple University, Philadelphia, USA; 2 University of Delaware, Newark, USA; 3 University of Connecticut, Storrs, USA; 4 NDRI, New York, USA; 5 National Institute on Drug Abuse, USA

**Keywords:** HIV, Correctional facility, NIATx, Change team

## Abstract

**Background:**

Persons held in correctional facilities are at high risk for HIV infection and their prevalence of HIV is substantially higher than in the general population. Thus, the need for proper surveillance and care of this high risk population is a paramount public health issue. This study aims to evaluate an organization-level intervention strategy for improving HIV services for persons in prison or jail.

**Methods/Design:**

HIV Services and Treatment Implementation in Corrections (HIV-STIC) is using a cluster randomized trial design to test an organization-level intervention designed to implement improvements in preventing, detecting, and treating HIV for persons under correctional supervision. Matched pairs of prison or jail facilities were randomized using a SAS algorithm. Facility staff members in both Experimental and Control conditions involved in HIV service delivery are recruited to receive training on HIV infection, the HIV services continuum, and relevant web-based resources. Staff members in both conditions are tasked to implement improvements in HIV prevention, testing, or treatment in their facility. In the Control condition facilities, staff participants use existing techniques for implementing improvement in a selected area of HIV services. In contrast, the Experimental condition staff participants work as a Local Change Team (LCT) with external coaching and use a structured process improvement approach to improve a selected part of the HIV services continuum. The intervention period is 10 months during which data are obtained using survey instruments administered to staff members and aggregate services delivery data. The study is being implemented in 13 pairs of correctional facilities across nine states in the US. Experimental sites are hypothesized to show improvements in both staff attitudes toward HIV services and the number and quality of HIV services provided for inmates.

**Discussion:**

The current study examines a range of process and outcome data relevant to the implementation of a Change Team approach across diverse correctional settings in the United States. This initial study represents an important step toward a national best practices approach to implementing change in U.S. correctional settings and could serve as an exemplar for designing similar implementation studies.

**Electronic supplementary material:**

The online version of this article (doi:10.1186/2194-7899-1-8) contains supplementary material, which is available to authorized users.

## Background

Prison inmates are at high risk for incident HIV infection and their prevalence of HIV is substantial, estimated at 1.5% of all inmates in federal or state custody at the end of 2010, making the need for proper surveillance and care a paramount public health issue (Maruschak 2012). Although many correctional facilities offer HIV testing and prevention services, including the provision of antiretroviral therapy (ART), numerous studies have demonstrated fundamental gaps in the delivery of effective HIV-focused care (Baillargeon et al. 2009; Beckwith et al. 2010; Springer & Altice 2005). Fewer than half of state prison systems offer opt-out testing (Centers for Disease Control and Prevention 2009), yet such testing protocols are cost effective and can help identify inmates with undetected infections (Begier et al. 2010). A number of barriers limit expansion of HIV testing among inmates, including stigma and discrimination (Earnshaw & Chaudoir 2009), timing of the tests, and lack of linkage with routine clinical exams (Kavasery et al. 2009).

Both primary and secondary interventions are important for this population. Primary HIV prevention is directed at all inmates, regardless of HIV status, and secondary HIV prevention is tailored to individuals who have an HIV infection. However, many inmates choose to not get tested regularly, testing is not offered routinely throughout an inmate’s incarceration term, HIV prevention interventions may not be evidence-based or appropriate for specific high-risk categories (e.g., injection drug users, women who engage in risky sex, Spanish-speaking inmates, female drug users), and primary and secondary prevention outcomes are not routinely assessed following release from prison (Centers for Disease Control and Prevention 2009; Hammett 2006). For inmates released to parole or under other types of community supervision, HIV prevention is not usually a priority, and access to HIV testing/counseling and prevention services is not routinely available (Beckwith et al. 2010; Springer & Altice 2005).

Perhaps an even greater concern, inmates identified as HIV-infected may be provided with ART by correctional facilities because of constitutional mandates for provision of inmate health care, but linkage to care following release can be problematic. There are typically disruptions in HIV-focused care once the inmate is released to the community. These disruptions may be caused by insufficient supply of medications provided by the facility at release, delays in obtaining Medicaid, and adherence problems by released inmates (Baillargeon et al. 2009), which can have serious health consequences for the HIV-infected inmate and the community (Springer et al. 2011). Interruptions in antiretroviral therapy can result in increased viral load or reduced effectiveness of the medications, and thus a heightened risk of HIV transmission (Deloria-Knoll et al. 2004; Mannheimer et al. 2002; Paterson et al. 2000). Barriers also exist for initiating and adhering to ART within correctional facilities, due to concerns about confidentiality, stigma, and lack of information (Earnshaw, & Chaudoir 2009; Roberson et al. 2009). Effective secondary prevention programs for HIV-infected inmates are also important for reducing the spread of infection to their sexual and drug-using partners. It is also common for community-based HIV service providers to encounter a range of difficulties as basic as gaining access to institutions to deliver services. For example, peer-based interventions that incorporate former inmates as facilitators may not be permitted in some correctional facilities. For both HIV-negative and HIV-infected inmates, improvements in pre-release planning/transitioning are needed. Because the time immediately following release from prison to the community is a particularly high-risk period, it is important to foster continuity of care approaches and secondary HIV prevention services (Gough et al. 2010). Several strategies for improving pre-release discharge planning have been identified; effective linkages need to include engagement in substance abuse or mental health treatment and housing, in addition to HIV medical care (Baillargeon et al. 2010a,2010b; Rich et al. 2001; Springer et al. 2011; Wolitski & Project START Writing Group 2006).

Improvements in the implementation and delivery of HIV-related services in correctional facilities are urgently needed to identify seropositive inmates, improve access to and utilization of HIV testing services, enhance continuity of prevention and medical care for inmates released to the community, and expand access to evidence-based prevention and ART for individuals under community supervision. Models of sexually transmitted disease transmission dynamics (Beckwith et al. 2010; Jürgens et al. 2011) suggest that reducing or preventing infections in core risk groups, such as inmates, can greatly reduce transmission of HIV throughout the community.

Theories of public health impact suggest that increasing the receipt of HIV services into an at-risk population would raise the overall positive public health impact, even without significantly increasing the effectiveness of the intervention—since public health impact is a product of an intervention’s effect size and the rate of utilization of the intervention (Tucker & Roth 2006). Thus, interventions to increase HIV testing and detection of unidentified infections, and increase continuity of ART, if implemented on a large scale - such as across multiple correctional systems – could be expected to convey tremendous public health benefits. The complex challenges of improving the delivery of health services such as HIV testing, prevention, and treatment within correctional settings suggests that organizational or systems-level interventions are needed to change practice and increase the use of evidence-based practices (Taxman & Belenko 2012).

The study described in this paper addresses three major aims designed to test the effectiveness of a local change team process improvement approach for: (1) *improving the perceived value* of HIV services among staff of correctional and community HIV organizations, (2) *increasing service penetration* for inmates infected by or at risk for HIV, and (3) *improving the quality* of HIV service delivery (e.g., improved ART adherence) for high risk or HIV-infected inmates. These aims are informed by the Proctor et al. conceptual model of implementation research distinguishing between *intervention* strategies, *implementation* strategies, and three levels of outcomes, including implementation, service, and client outcomes (Proctor et al. 2009). This implementation model proposes that implementation strategies can target one or more of the five levels of the service delivery environment, including individual providers, supervisory practices, group learning, and organizational and systems environments. We examine the effectiveness of implementation strategies at the organizational levels described by this model. In this model, improvements in client outcomes are viewed as dependent not only on evidence-based practices and programs, including client factors such as adherence to treatment, but also on whether the innovation is an improvement to existing practices. Service outcomes and by extension, client outcomes, are viewed as dependent on the quality of the implementation itself. The implementation model is based on the assumption that successful implementation will result in improved service outcomes which, in turn, will lead to enhanced client outcomes. As such, two types of outcomes are being assessed in the HIV-STIC study: (1) Implementation outcomes and (2) Service-level outcomes.

Three primary hypotheses are being tested:

Hypothesis 1: Value. Compared to the Control condition, staff members from facilities in the Experimental condition show greater improvements in their ratings of the value of implementing HIV services.

Hypothesis 2: Services Penetration. Compared to the Control condition, proportionately more inmates in the Experimental condition who are at risk of or infected by HIV receive services within the HIV services continuum.

Hypothesis 3: Quality of Service Delivery. Compared to the Control condition, proportionately more inmates in the Experimental condition who are infected by or at risk for HIV receive improved services delivery within the HIV services continuum.

### Methods/Design

#### Study design

The HIV Services and Treatment Implementation in Corrections (HIV-STIC) study evaluates the experimental condition of using a modified Network for the Improvement of Addiction Treatment (NIATx) model (McCarty et al. 2007), which has been applied successfully in drug abuse treatment facilities (Hoffman et al. 2008), to improve HIV services in criminal justice settings, specifically correctional (i.e., jail or prison) facilities. The NIATx organizational change approach utilizes the program administrator as the Executive Sponsor of a Local Change Team (LCT) consisting of a Change Leader who has access to the Executive Sponsor and members agreed upon by the Change Leader. The NIATx approach focuses on improving access to services and retention in treatment (McCarty et al. 2007; Capoccia et al. 2007). It incorporates five principles to identify problems and introduce and test organizational changes: (1) understand and involve the customer, (2) fix key, important problems, (3) pick a powerful change leader, (4) get ideas from outside the organization, and (5) use rapid cycle testing (McCarty et al. 2009). Each of these principles is articulated, well-defined, and reflected in structured activities that contribute toward identified organizational goals.

In the current study, a modified NIATx approach is being compared to a conventional HIV staff training approach to improve the delivery of the continuum of HIV services to jail or prison inmates. HIV services include routine HIV testing, prevention/education programming, and procedures to link HIV-infected individuals to community-based treatment after confinement. A non-blinded cluster randomized design is used with 14 pairs of correctional facilities randomized within nine participating study sites in the US. Some of the study sites have one pair of facilities in the study while other sites have two pairs. The facilities are matched as pairs, with the basic characteristics of each pair matched as closely as possible to ensure an equivalent chance of successful outcomes, based on size of inmate population and custody/classification levels (e.g., minimum or medium). One facility in the pair is randomly assigned to the Control Condition while the other is assigned to the Experimental Condition.

The quality improvement process tested in HIV-STIC is modeled after the NIATx approach, but differs in important respects. Notably, the goal of the HIV-STIC is to improve HIV testing and linkage to treatment, rather than drug abuse treatment access and utilization. The study also spans across organizations (correctional agencies, community health and drug abuse treatment agencies), which places greater emphasis on cross-agency collaboration and coordination.

#### Study conditions

An initial face-to-face stakeholder orientation meeting is designed to bring together criminal justice senior management and the research center (RC) Principal Investigator (PI)/researcher for an introduction to the HIV-STIC study and overview of the study protocol in each of the nine study sites. The RC PI designates an Executive Sponsor for the study who is a senior agency administrator. Across the nine study sites, Executive Sponsors include state-level managers of correctional health programs, medical services directors, and general program directors. Topics for discussion at the orientation meeting include an overview of the HIV-STIC and its goals, as well as details about study components , including timeline, baseline training, randomization, staff participation, consent process, and data collection. Based on the discussions at this meeting, the Executive Sponsor selects one of the components of the HIV services continuum – prevention/education, testing, or linkage to treatment -- on which to focus in the correctional agency selected for the study. The Executive Sponsor also selects Facility Sponsors (see Table 1) for each of the study facilities. Additional study participants are then selected by the Executive and Facility Sponsors based on their involvement in delivering HIV services at the facilities; these staff participate in baseline training prior to study site randomization.

**Table 1:** **Participant roles in HIV-STIC**

Executive Sponsor	An agency-level administrator who determines which area of the HIV services continuum will be the primary focus for all facilities in their state or county. Monitors progress at all sites but is not involved in day-to-day management of the implementation and change process.
Facility Sponsor	In a position of senior authority at the prison or jail facility responsible for the overall change process. Authorizes the staff time and resources at the prison or jail facility needed to complete the project and participates with the Change Team Leader in selecting the change team members. Closely follows the progress of the LCT; occasionally participates in team meetings; and meets frequently with Change Team Leader. Selected by the Executive Sponsor.
Change Team Leader	Interacts with the Facility Sponsor, keeping the Facility Sponsor informed of the change team progress, requests the resources needed to accomplish the change team goal, works with the LCT Coach, tracking the progress of the change team against the study timelines, calls meetings of the change team, and assigns tasks and roles within the team. Selected by the Facility Sponsor.
Change Team Coach	An external consultant trained in NIATx process improvement strategies. The Coach works with Change Team Leader and agency representatives to help LCT identify roadblocks and other issues which may impede effective implementation. The Coach spends one day training the change team and conducts regular and in-person coaching sessions with the Change Team Leader and LCT. The Coaches from all study sites participate in a collective monthly call with a mentor who is a senior member of the NIATx core organization. Selected by the Researcher.
Local Change Team	Consists of about 5–7 staff members from facility units affected by changes in HIV service delivery or agencies related to or affected by any changes, including prison medical staff, program staff, counselors, and community-based HIV service coordinators. The Team may also include an outside expert in the field of HIV services.

#### Baseline training

This training is designed to provide basic information and resources relevant to the HIV services continuum as well as evidence-based practices for implementing HIV services to criminal justice populations. The content of this training includes the HIV services continuum, HIV prevalence and issues among inmates, and evidence-based HIV services in institutional and community corrections. Staff attending the training include the Executive Sponsor, the Facility Sponsors, the facility medical directors, health care staff involved with HIV testing, HIV counselors, qualified HIV interventionists, prison/jail pharmacists, the drug treatment coordinator, drug treatment staff, and corrections officer supervisors, as well as participants from identified community-based treatment and health organizations providing HIV-related care to newly released prisoners. One joint training session involving staff of the two or four facilities selected for the study is held for each of the study sites. Thus, nine baseline trainings are conducted. Following the training, informed consent is administered to the staff participants and the baseline surveys administered.

The baseline training lasts about six hours and utilizes adult learning principles which include connecting with personal experiences, creating a safe learning environment, accommodating various learning styles, and providing active learning activities on HIV. The curriculum focuses on knowledge acquisition (lecture, discussion, review of printed materials) as well as skills training achieved through such activities as role-playing exercises involving realistic scenarios and practice sessions. The training also includes a review of evidence-based HIV prevention programs and information from the Centers for Disease Control and Prevention’s Diffusion of Effective Behavioral Interventions project (Solomon et al. 2006); antiretroviral therapy and adherence; HIV testing and counseling procedures and relevant policies, and pre- and post-release planning models. Local policies and issues relevant to the focus area at each Department of Corrections (DOC) are incorporated and discussed in training segments on implementation issues. The training is interactive and action-oriented, and is designed to assist site staff to identify key agencies/staff positioned to deliver improved HIV services. Following baseline training, the matched pairs of study sites are randomized into two study conditions. Randomization was conducted by the chair of the CJDATS Steering Committee using a SAS algorithm.

#### Control condition

**Figure 1:**
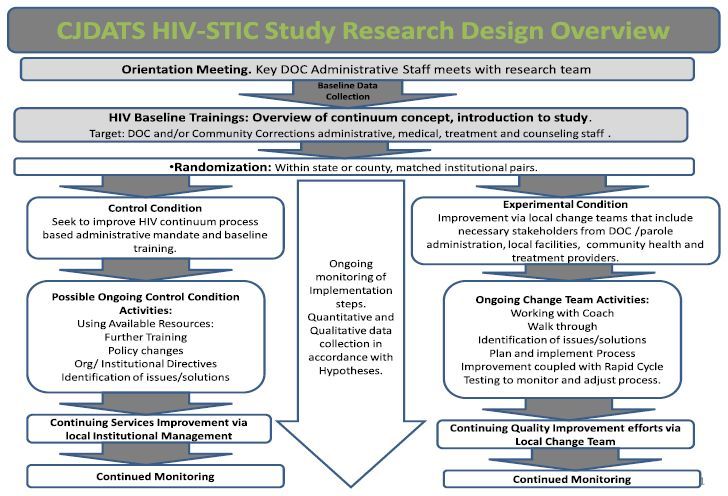
**Overview of HIV-STIC study design.**

In addition to the standardized baseline training for all sites, participants at the control condition sites are provided web-based informational resources regarding a range of relevant HIV-focused care services. An individual who holds a management or supervisory position in the facility is designated as a Facility Sponsor and selected by correctional agency management to implement improvement(s) to the HIV service delivery continuum at the control facilities. The leadership of those sites that focus on linkage to care may include a representative from the targeted community treatment agency. Management engages additional staff at different stages of the study to work on HIV services improvements (see Figure 1).

#### Experimental condition

Participants at the experimental condition sites are involved in an approach to implementation that requires the creation of local change teams (LCT) that work toward identifying and accomplishing specific process improvement goals with regard to HIV-focused care. Each LCT consists of 5–7 permanent members, most of whom are from the correctional agency (prison or jail; see Table 1) or the agency’s contracted on-site medical provider. Eligible change team members have direct responsibility for or direct involvement with the selected area of the HIV services continuum and/or the specific evidence-based practice being implemented at a site. Preference is given to staff with responsibilities that affect the delivery of HIV services who are recommended by correctional agency management and who are expected to remain in their current position for the duration of the study. For sites that are focused on linking inmates to post-release HIV-related care, a community-based HIV service provider is also identified.

Experimental Condition participants engage in process improvement strategies that involve a structured set of activities collectively designed to produce organizational change (McCarty et al. 2007). The assumption underlying this approach is that creating change in organizational operations requires a sustained multi-level effort that involves leadership, ongoing staff involvement, understanding the organization’s function from multiple viewpoints including the “consumer” of services, systematic efforts to test and measure new practices or interventions within the organization, and thoughtful decision-making regarding the results of the process improvement efforts. Initiation of the modified NIATx process involves several distinct roles; the primary participants are Executive Sponsor, Facility Sponsor, Change Team Leader, and a Change Team Coach. Short descriptions of these roles appear in Table 1.

The initial task of the LCT is to refine the problem initially identified by the Executive Sponsor and the Facility Sponsor, and to reduce the problem into components that can be individually addressed. Two procedures adapted from NIATx are employed to gain a better understanding of the problem and its component parts. A walk-through is conducted in which two or more members of the LCT go through the same process as the inmate regarding the specific service under scrutiny. Confidential or anonymous feedback is solicited from staff involved in the process based on their observations and experience. The walk-through and staff observations are then reviewed in order to identify barriers within the system. The team then chooses a single manageable component to be changed, followed by additional components as each change is successfully implemented, accepted, or revised. Examples of process improvement goals that the LCT teams have identified include:

-  Increase female attendance at HIV prevention sessions;
-  Increase percentage of inmates receiving HIV prevention/education in 60-day period before release;
-  Increase percentage of inmates receiving HIV test at admission;
-  Increase HIV testing and;
-  Improve linkage to community treatment for HIV + inmates.

The implementation process involves rapid cycle testing with teams following a Plan, Do, Study, Act (PDSA) process. The frequency of team meetings is expected to vary between study sites, but generally takes place bi-weekly or monthly. Baseline data are collected for 6 weeks prior to implementing the changes. These are not research data, but rather data assembled by the LCT to inform its work. The change is then implemented and data are collected for a period of 3–6 weeks. Generally, data consist of simple aggregates that can be shown on a single line graph so as to make progress easy to interpret and demonstrate to the agency staff. If the change is unsuccessful, another change is substituted. If the change is successful, an additional change is made to further improve the process. After successful changes, the team formulates a sustainability plan and responsibility for following up on the changes is assigned to one of the change team members. In some cases, a new sustainability team is formed along the same principles as the original change team.

### Participating sites

Six of the nine study sites implement the HIV-STIC study in state prison facilities where inmates transition to the community under parole supervision after release; however, three study sites implement HIV-STIC in jail facilities where inmates have pending court cases or short sentences. Study sites focusing on linking exiting inmates to community-based HIV services identify at least one collaborating community-based program that provides HIV services and medications to be involved in the study. To the extent possible, the correctional and community-based agencies involved in HIV-STIC are autonomous such that introduction of the intervention in one agency does not contaminate process improvements surrounding HIV services in other agencies. Separate adult correctional institutions are considered independent organizations if they do not share a common central administrative entity that is responsible for the establishment and promulgation of agency policies and procedures regarding within-facility delivery of HIV services. The facility is the potential target of the organizational intervention.

Administrators of the correctional facilities selected for the study and corresponding administrators of HIV community services organizations select staff to participate in the HIV-STIC study, including staff responsible for prevention, testing, and treatment activities. These staff members vary as needed by site, but include HIV counselors, prison medical staff, substance abuse treatment staff, and community-based HIV services staff. Staff member participants are asked to complete surveys, participate in semi-structured interviews, and adhere to study protocols. Additionally, staff member participants in the experimental condition serve as members of the LCT.

### Data collection and measures

Primary and secondary outcomes for the study were selected based on both the implementation research framework suggested by Proctor and her colleagues (Proctor et al. 2009) and the public health impact framework. The primary sources of data for this study are staff surveys, participating agency records of services, and anonymous inmate surveys.

#### Primary outcomes

The three primary outcomes are *value, services penetration, and services quality*.

##### Value

In this study, value is defined as a combination of the acceptability, perceived feasibility, and perceived relative costs of implementing HIV service improvements as perceived by staff members involved in the delivery of HIV services in the study sites. One measure of value is a set of items adapted from the Barriers to Research Utilization Scale (Funk et al. 1991). This instrument contains subscales for characteristics of the adopter, characteristics of the organization, characteristics of the innovation, and characteristics of the communication. Another measure, a modified version of the Usage Rating Profile-Intervention (Chafouleas et al. 2009), focuses more specifically on the acceptability and perceived relative costs of implementing enhancements of HIV services. This instrument contains subscales for acceptability, knowledge, feasibility, and systems support. Feasibility is also measured using a modified version of the Evidence Based Practice Attitudes Scale (Aarons 2004), which includes subscales for requirements, appeal, openness and divergence. All scales are taken from previous studies and have good psychometric properties.

##### Services penetration

Services penetration is defined as the extent to which an evidence-based HIV service reaches the appropriate target population. An example is the proportion of inmates that receive HIV tests at admission. Because several study sites have very few staff involved in HIV testing or linkage to treatment in the community, penetration does not focus on staff measures, but rather focuses on the measure of penetration as the proportion of inmates who receive an HIV service such as HIV testing, prevention intervention, or linkage of HIV-infected inmates to treatment in the community. The services penetration measures are collected at the aggregate inmate level.

##### Quality of service delivery

Improvements in the quality in which HIV services are delivered in correctional facilities are related to the service-level outcomes of efficiency, effectiveness, or timeliness of the service delivery. Outcomes linked directly to these improvements include time between assessment and HIV testing, increases in the number agreeing to an HIV test, increases in the number of inmates who receive ART, and improved HIV treatment continuation rates.

#### Secondary outcomes

Secondary outcomes are stigma, interagency collaboration, and inmate awareness.

##### Stigma

Stigma may be related to two implementation outcome measures: (1) whether HIV service improvements are perceived as having value and (2) the extent of acceptance of HIV service improvements by correctional staff. An adapted version of the HIV/AIDS Stigma Scale (Zelaya et al. 2008) is given to staff in the study sites. Embedded within the 21 items of this instrument are 4 subscales reflecting fear of transmission and disease, association with shame, blame and judgment, personal support of discriminatory actions or policies, and perceived community support of discriminatory actions or policies.

##### Interagency collaboration

Cooperation and coordination are described as key determinants of process improvement interventions targeted at the group or team level (Proctor et al. 2009). Thus, interagency coordination and collaboration are expected to be key factors determining implementation outcomes at the organizational and systems (defined as cross-organizational) levels. Improvements in HIV services, especially as inmates transition to the community, are expected to result in experimental site increases in interagency contacts, communication, program development, cross-agency training, client service activities, and changes in collaborative policies. These changes are expected to occur as part of changes in program/service delivery processes that are implemented as a part of the LCT intervention. Interagency collaboration is being measured with a modified version of the Interagency Collaborative Activities Scale (Dedrick & Greenbaum 2011) and semi-structured interviews with facility site administrators and key service staff.

##### Inmate awareness

Although the study’s main focus is the organizational system, a process improvement project designed to focus on the delivery of HIV services ought to impact the individuals receiving those services. As such, an additional outcome is the perceived value of services based on the perceptions of the inmates themselves. An anonymous survey of inmates at the study sites includes questions about each of the areas of the HIV services continuum (education and prevention, testing, treatment), as well as pre-release planning.

### Data sources

Staff surveys are used to capture data related to value, stigma and interagency collaboration, as well as measure staff perceptions of the characteristics of the organization, and are completed prior to the baseline training and 10 months after the experimental site kick-off meeting. Thus, the Barriers to Research Utilization, EBPAS, HIV Stigma and Interagency Collaboration scales are completed at baseline, prior to training. The modified Usage Rating Profile-Intervention and the TCU-Workshop Evaluation Form (Bartholomew et al. 2007) are completed immediately after the baseline training. Ten to fifteen staff members at both Experimental and Control sites are selected to receive the surveys, including nurses, correctional officers, substance abuse treatment counselors, deputy wardens, case managers, pre-release discharge counselors, nursing administrators, and community HIV staff such as nurses and administrators. At the end of the study, staff members also complete a short survey designed to assess the impact of the change team process on the facility, including staff acceptance of the change process and whether sufficient training resources exist to support modifications initiated by the change team. Finally, members of the change team in the Experimental sites complete a short survey on the impact of the change team process.

Participating agency records are used to collect services penetration and quality of service delivery data related to the process improvement measures for HIV prevention/education, testing, and treatment. These data are collected at monthly intervals beginning with the initiation of the intervention at the kick-off meeting and concluding two months after the Experimental intervention ends for a total of 12 months. The nine primary HIV service penetration and quality of service items that are used in the analysis are presented in Table 2.

**Table 2:** **HIV-STIC service penetration and quality of service items**

***Prevention and Testing***	***Linkage to Antiretroviral (ARV) medication in the Community***
Percent of inmates who receive the HIV prevention intervention	Percent of inmates given an appointment with a community HIV treatment provider
Percent of inmates who complete the HIV prevention intervention	Of those with appointment, percent who contacted community HIV treatment provider
Percent of inmates who receive an HIV test	Percent assigned a case manager or linkage coordinator
Time between the HIV test and results counseling	Percent discharged with supply of ARV medication
	Of those discharged with ARV prescription, percent who refill the prescription

The anonymous survey of inmates is administered in both Experimental and Control sites as a means of assessing awareness of and attitudes toward the HIV services continuum among the general inmate population at each study site. It provides a secondary measure of penetration and value—from the perspective of the inmate—of the HIV service (prevention/education, testing, and/or treatment) that is the focus of the study site. Prior to the start of the study and at the end of the final data collection phase, at each facility, the research team administers the survey to a convenience sample of a minimum of 50 inmates per facility. Data are anonymous and are not collected longitudinally.

In addition to the quantitative data collection, qualitative data are collected in both Experimental and Control sites in the form of in-depth interviews with selected staff who are involved in the intervention and one focus group with the eight coaches assigned to the change teams (one coach is working with two study sites). The qualitative data provide additional contextual information to complement the quantitative data collected. In effect, the qualitative data help to explain why certain quantitative outcomes emerge in the patterns observed. In order to accomplish this, several themes are developed from each of the primary and secondary hypotheses. For example, the theme ‘perceived importance of service improvement’ emerges from primary hypothesis 1 (Value). Specific interview questions are constructed from each theme that, in turn, map onto the hypothesis. For example, the following is asked of the LCT members: “In your opinion, how important are the goal(s) that the Change Team decided to address? Tell me some more about that please. Do you feel that other goals should also be addressed?” These questions and others in the interview guide ask for staff views about the perceived importance of service improvement related to HIV services and thus provide detailed explanatory information regarding hypothesis 1 (Value). The same process is done for each primary and secondary hypothesis.

The focus group with the coaches provides additional contextual information regarding their experience in implementing a process improvement approach in correctional settings. The primary aims of the focus group are to understand: 1) the challenges as a coach for implementing a process improvement approach in correctional settings, and 2) the challenges as a coach for implementing a process improvement approach as part of a research project with an experimental design.

In order to monitor fidelity to the intervention and gather information about site activities such as changes in personnel, administration, or policies that may affect the intervention, site research teams complete a quarterly assessment of fidelity in the Experimental sites and a monthly site activity assessment in both Experimental and Control sites. The monthly site activity data collection also captures changes in the local environment unrelated to the study that may affect study outcomes.

Finally, data are also collected to estimate the costs associated with the intervention. The cost to an organization of implementing a process improvement strategy is a potential barrier for sustainability. Costs vary depending on the particular HIV services goal at each site and particular barriers faced in their systems. Because of the complexity and variation across states and study sites in costs of HIV medications and HIV tests, it is not feasible to do a full-scale cost analysis. For example, because the costs of antiretroviral therapy differ, and prescriptions depend on client- and state-specific guidelines and protocols, it would be necessary to gain access to individual prescription data and update these data if prescriptions change over time. Such data collection efforts are not feasible for this study. A benefit-cost analysis is also not possible because individual client outcome data are not collected for this study. Nonetheless, it is useful to collect some cost data because intervention costs may affect the likelihood of replication. Thus, study sites utilize a brief form to collect data on the amount of time that staff members spend on study-related activities. In addition, the costs of HIV tests at each study site are collected to assess the costs associated with any increased testing as a result of the intervention.

### Timeline

In this implementation study, we seek to test Hypotheses 1–3 that an organization-level intervention strategy can improve the delivery of HIV services for preventing, detecting, and treating HIV for offenders under correctional supervision. The duration of the intervention period is 10 months during which data are obtained using a battery of survey instruments, and aggregate services delivery data, as described above. Information is collected at baseline using the baseline survey of organizational characteristics (BSOC), HIV Staff Survey (comprised of four scales), HIV Services Delivery (modified Usage Rating Profile-Intervention), Workshop Evaluation Form (WEVAL), and Inmate survey. At the 10-month follow-up point the same instruments are used as well as the Facility Impact Assessment, Change Team Assessment, and qualitative interviews. The BSOC and the WEVAL are only collected at baseline. Finally, we collect information on a monthly basis – over a 12 month period – with regard to agency records, cost data, and site activity. Thus, these data collectively allow us to test Hypotheses 1–3 pertaining to the impact of an organization-level intervention strategy. The data collection plan for the study is presented in Table 3. All sites except one were in the field with the intervention by early 2012, with one site initiating the protocol in summer 2012. Data collection will end in early 2013 and initial results are expected in late 2013.

**Table 3:** **HIV-STIC data collection plan**

Instrument	Baseline	Immediately post training	6 mths	10 mths
BSOC	X			
HIV Staff Survey (4 scales)				
Barriers to Research Utilization	X		X	X
EBPAS	X		X	X
Stigma	X		X	X
Interagency Collaboration	X		X	X
HIV Services Delivery		X	X	X
TCU WEVAL		X		
Inmate Survey	X			X
Qualitative Interviews			X	X
Facility Impact Assessment				X
Change Team Assessment				X
Agency Records		X*	X	X
Cost Data		X*	X	X
Site Activity Report		X*	X	X

*Aggregate data collected every month beginning with the month following training and continuing for 12 months.

## Discussion

Although evidence-based practices for HIV services in correctional settings have been identified, successfully moving these practices into routine and efficacious use in the field is an important challenge. Implementation research is needed to determine the optimal strategies for achieving this goal (Rubenstein & Pugh 2006). Improving implementation, and ultimately the sustainability, of health services and evidence-based practices requires careful attention to the systems, organizational, and staff contexts within which these services are delivered (Proctor et al. 2009). The multifaceted nature of these contexts suggest that unless various staff members embrace the need for and value of service improvements, such improvements are not likely to be well implemented or sustained.

Epidemiological models of sexually transmitted disease transmission and public health models for improving public health impact of interventions indicate that public health impacts are maximized by detecting and reducing infections within high-risk groups, and increasing the utilization of prevention and treatment services (Tucker & Roth 2006; Anderson 1991; Aral 2002; Blanchard 2002). Yet there has been little rigorous research on the implementation of HIV services, or strategies for improving implementation outcomes (especially staff perceptions of the services) in correctional settings.

The high rate of HIV infection among incarcerated persons calls for interventions that are effective in various correctional settings in the US. The dual priorities of health and security in correctional settings require staff from different departments or organizations to work across their usual domains in order to provide adequate services. The HIV-STIC study is designed to test whether a process improvement model based on the utilization of Local Change Teams can significantly improve services across the full continuum of HIV care, including prevention, testing, and linkage to care upon release. The study design described in this paper will enable the research team to investigate a wide range of process and outcome data relevant to the implementation of the Change Team model across diverse correctional settings in the US. It is hoped that this study will be an important step toward a national best practices approach to implementing change in correctional settings and may also serve as an exemplar for similar implementation studies in other areas of health services for correctional populations and settings.

## Authors’ original submitted files for images

Below are the links to the authors’ original submitted files for images. Authors’ original file for figure 1
