# Supplementary material for: A cluster randomized trial of utilizing a local change team approach to improve the delivery of HIV services in correctional settings: study protocol
Source: Health Justice. 2013 Dec 23;1:8. doi: 10.1186/2194-7899-1-8 (PMC4270366; doi:10.1186/2194-7899-1-8)
Supplement: Supplementary file 1 — Authors’ original file for figure 1 [file 40352_2013_5_MOESM1_ESM.pdf]

CJDATS HIV-STIC Study Research Design Overview

Orientation Meeting. Key DOC Administrative Staff meets with research team

Baseline Data Collection

HIV Baseline Trainings: Overview of continuum concept, introduction to study.
Target: DOC and/or Community Corrections administrative, medical, treatment and counseling staff .

•**Randomization:** Within state or county, matched institutional pairs.

Control Condition
Seek to improve HIV continuum process based administrative mandate and baseline training.

Possible Ongoing Control Condition Activities:
Using Available Resources:
Further Training
Policy changes
Org/ Institutional Directives
Identification of issues/solutions

Continuing Services Improvement via local Institutional Management

Continued Monitoring

Ongoing monitoring of Implementation steps.
Quantitative and Qualitative data collection in accordance with Hypotheses.

Experimental Condition
Improvement via local change teams that include necessary stakeholders from DOC /parole administration, local facilities, community health and treatment providers.

Ongoing Change Team Activities:
Working with Coach
Walk through
Identification of issues/solutions
Plan and implement Process Improvement coupled with Rapid Cycle Testing to monitor and adjust process.

Continuing Quality Improvement efforts via Local Change Team

Continued Monitoring
